# The heterologous expression of *Escherichia coli* MutT enzyme is involved in the protection against oxidative stress in *Leishmania braziliensis*

**DOI:** 10.1590/0074-02760190469

**Published:** 2020-07-06

**Authors:** Laila de Carvalho Andrade, Ana Maria Murta Santi, Ceres Luciana Alves, Wesley Roger Rodrigues Ferreira, Antônio Vinícius de Assis, Edward Oliveira, Carlos Renato Machado, Silvane Maria Fonseca Murta

**Affiliations:** 1 Fundação Oswaldo Cruz-Fiocruz, Instituto René Rachou, Belo Horizonte, MG, Brasil; 2 Universidade Federal de Minas Gerais, Instituto de Ciências Biológicas, Departamento de Bioquímica e Imunologia, Belo Horizonte, MG, Brasil

**Keywords:** Leishmania braziliensis, Leishmania infantum, MutT, DNA repair, 8-oxoG, oxidative stress

## Abstract

**BACKGROUND:**

Oxidative stress is responsible for generating DNA lesions and the 8-oxoguanine (8-oxoG) is the most commonly lesion found in DNA damage. When this base is incorporated during DNA replication, it could generate double-strand DNA breaks and cellular death. MutT enzyme hydrolyzes the 8-oxoG from the nucleotide pool, preventing its incorporation during DNA replication.

**OBJECTIVES:**

To investigate the importance of 8-oxoG in *Leishmania infantum* and *L. braziliensis*, in this study we analysed the impact of heterologous expression of *Escherichia coli* MutT (EcMutT) enzyme in drug-resistance phenotype and defense against oxidative stress.

**METHODS:**

Comparative analysis of *L. braziliensis* and *L. infantum* H_2_O_2_ tolerance and cell cycle profile were performed. Lines of *L. braziliensis* and *L. infantum* expressing EcMutT were generated and evaluated using susceptibility tests to H_2_O_2_ and Sb^III^, cell cycle analysis, γH2A western blotting, and BrdU native detection assay.

**FINDINGS:**

Comparative analysis of tolerance to oxidative stress generated by H_2_O_2_ showed that *L. infantum* is more tolerant to exogenous H_2_O_2_ than *L. braziliensis.* In addition, cell cycle analysis showed that *L. infantum*, after treatment with H_2_O_2_, remains in G1 phase, returning to its normal growth rate after 72 h. In contrast, after treatment with H_2_O_2_, *L. braziliensis* parasites continue to move to the next stages of the cell cycle. Expression of the *E. coli* MutT gene in *L. braziliensis* and *L. infantum* does not interfere in parasite growth or in susceptibility to Sb^III^. Interestingly, we observed that *L. braziliensis* EcMutT-expressing clones were more tolerant to H_2_O_2_ treatment, presented lower activation of γH2A, a biomarker of genotoxic stress, and lower replication stress than its parental non-transfected parasites. In contrast, the EcMutT is not involved in protection against oxidative stress generated by H_2_O_2_ in *L. infantum*.

**MAIN CONCLUSIONS:**

Our results showed that 8-oxoG clearance in *L. braziliensis* is important to avoid misincorporation during DNA replication after oxidative stress generated by H_2_O_2_.

Leishmaniasis refers to a spectrum of diseases caused by protozoan *Leishmania* parasites. It is a neglected tropical disease and represents one of the major public health problems in developing countries of the Indian subcontinent, South-East Asia, Latin America and East Africa according to World Health Organization.^1^ Human leishmaniasis has a prevalence of 12 million cases and an incidence of 1.2 million new cases annually, with an estimated population of 350 million at risk.^2^ Depending on genetic and environmental factors, the host immune response and mainly on *Leishmania* species involved, the disease can comprise two main clinical forms: visceral leishmaniasis or cutaneous leishmaniasis. In the New World, *L. (Viannia) braziliensis* is the causative agent of cutaneous and mucocutaneous leishmaniasis, whereas *L. (Leishmania) infantum* (syn. *L. (L.) chagasi*) causes visceral leishmaniasis, which is lethal if not treated.^3^

In the absence of an efficient vaccine for human, the control of leishmaniasis relies primarily on chemotherapy. Pentavalent antimony-containing compounds such as sodium stibogluconate (Pentostam^®^) and N-methyl-glucamine (Glucantime^®^) have been used as first-line therapies against all forms of leishmaniasis. The mechanisms of action of antimony are not entirely clear. It is generally agreed that trivalent antimony (Sb^III^) is the active form of the drug against amastigote and promastigote forms of *Leishmania*.^4^ Some studies have suggested that antimony inhibits macromolecule biosynthesis in amastigotes, possibly via inhibition of glycolysis and fatty acid oxidation.^5^ Earlier report has indicated that antimonials cause perturbations in the thiol redox potential, which would drive to parasite death by oxidative stress.^6^ Trypanosomatids are constantly exposed to different reactive oxygen species (ROS), such as superoxide anion, hydrogen peroxide and hydroxyl radicals, formed during the host defense reaction, either by exogenous (chemotherapeutic) or endogenous action (metabolism of the parasite itself or inflammatory cytokines). The action of ROS can lead to deleterious damage to DNA of the parasite and hence its death. Thus, an effective antioxidant defense system, which can prevent and repairing the damage caused by oxidising agents, is essential for the survival of the parasite inside parasitophorous vacuoles in human macrophages.

Oxidation represents the main endogenous factor of DNA damage, which can cause genomic instability and cell death. Due to its low redox potential, guanine (G) is the most vulnerable base to ROS oxidation.^7^ The main product of G oxidation is 7,8-dihydro-8-oxoguanine (8-oxoG).^8^ If not repaired during DNA replication, 8-oxoG can pair with adenine and cause a G:C to T:A transversion. During the DNA repair of 8-oxoG, double-strand breaks could be generated, which can be highly deleterious.^9^ Due to its high ability to cause lesions in the DNA, 8-oxoG is used as a cellular biomarker of oxidative stress.^10^

The GO-system is a three-component 8-oxoG repair pathway compost by genes MutT, MutY and MutM in bacteria and MTH1, MUTYH and OGG1 in humans, respectively.^11^ The enzyme MutT (or MTH1), focus of our study, hydrolyzes 8-oxo-dGTP in the nucleotide pool, returning it to the monophosphate form.^12^ The enzymes MutM (or OGG1) and MutY (MUTYH) are responsible for repairing 8-oxoG paired with cytosine in the DNA or removing the adenine in the 8-oxoG:A mispair, respectively.^7^ ^,^ ^8^

The MutT enzyme gene from *Escherichia coli* (NCBI: P08337) has 390 bp and encodes a protein with 129 amino acids. It belongs to the superfamily of Nudix Hydrolases (Nucleosides Diphosphates attached to other moieties, X). This superfamily contains a sequence called Nudix Box with a conserved 23-amino acid sequence GX_5_EX_7_REUXEEXGU, where X can be any residue and U is a hydrophobic residue.^13^

The leishmanial genomic project identified several DNA repair pathway genes in parasite genome.^14^ ^,^ ^15^ However, some important elements of the DNA repair machinery, such as a MutT homolog, have not yet been characterised. The complete sequence of the *MutT* gene in *Leishmania* spp. is available in database (TritrypDB). It is present in 13 different species of *Leishmania* as *L. braziliensis*, *L. infantum*, *L. major*, *L. aethiopica*, *L. donovani*, *L*. *mexicana*, *L. tarentolae*, *L enriettii*, *L. gerbii*, *L. arabica*, *L. panamensis*, *L. tropica* and *L. turanica* (TritrypDB). However, the role of the MutT enzyme in *Leishmania* has not been described yet.

In this study, we analyse the tolerance of *L. braziliensis* and *L. infantum* parasites to oxidative stress generated by H_2_O_2_ and compare their cell cycle after H_2_O_2_ treatment. In order to investigate the importance of 8-oxoG to oxidative stress we generated *L. braziliensis* and *L. infantum* parasites heterologously expressing *E. coli* MutT, since the role of this enzyme was well established. We analysed the phenotype these parasites in relation to growth in culture medium, tolerance to oxidative stress generated by H_2_O_2_, susceptibility to Sb^III^, cell cycle progression, DNA damage and replication stress generation.

## MATERIALS AND METHODS

*Leishmania spp. samples* - Promastigote forms of *L.* (*Viannia*) *braziliensis* (MHOM/BR/75/M2904) and *L. (Leishmania) infantum* (syn. *L. (L.) chagasi*) (MCAN/BR/2000/BH400) were used in this study. Parasites were grown at 26ºC in 199 medium (M199) supplemented with 2 mM L-glutamine, 5 μg/mL hemin, 50 μg/mL streptomycin, 2 μg/mL biopterin, 1 μg/mL biotin, 40 mM HEPES pH 7.4, 500 U penicillin and 10% v/v heat-inactivated fetal calf serum.^16^ These parasites were harvested in the logarithmic growth phase washed in phosphate buffered saline (PBS) (137 mM NaCl, 2.7 mM KCl, 10 mM Na_2_HPO_4_ and 2 mM KH_2_PO_4_) and the parasitic pellets were used for DNA and RNA preparations.

*Generation of E. coli mutT expressing L. braziliensis and L. infantum lines* - A 390 bp fragment corresponding to *E. coli mutT* encoding region (NCBI accession number: P08337) was amplified with *Pfx* DNA polymerase (Invitrogen, Life technologies, CA, USA) from AB1157 *E. coli* genomic DNA using the forward primer: 5’-tAGATCTccaccATGAAAAAGCTGCAAATTGC-3’ and the reverse primer: 5’-ttAGATCTCTACAGACGCTTAAGCTTCGCA-3’. The lower case letters indicate the Kosak sequence and the underlined sequences correspond to *Bgl*II restriction site. The obtained polymerase chain reaction (PCR) products were cloned into the pGEM-T Easy^®^ vector (Promega, Madison, WI, USA) and subsequently submitted to sequencing reaction for confirmation of correct sequence. All constructs were sequenced in an ABI 3130 (Applied Biosystems, Foster City, CA, USA). The pGEM-*EcmutT* constructs were restricted with *Bgl*II and the fragments released were subcloned into the dephosphorylated pIR1BSD expression vector (kindly provided by Dr Stephen Beverley, Washington University, USA). To confirm the correct direction of cloning, the constructs were then digested with *Hind*III and *EcoR*I separated releasing fragments that confirmed the sense direction of the *EcmutT* gene. Thus, the constructs pIR1BSD (empty vector), and pIR1BSD-*EcmutT* were linearised by *Swa*I digestion and electroporated into wild-type *L. braziliensis* and *L. infantum* lines using a Gene Pulser XCell electroporation system (Bio-Rad, Hercules, CA, USA) according to the protocol described by Robinson and Beverley.^17^ This stable transfection allowed integration of the pIR1 vector into the rDNA 18S ribosomal small subunit locus of the parasite.^17^ Colonies were obtained following plating on semisolid M199 containing blasticidin (BSD) (10 µg/mL). After 1-2 weeks, clonal lines were selected, and the presence of constructs was confirmed by PCR tests using genomic DNA with specific primers for the BSD marker.

*RNA purification and reverse transcription-PCR (RT-PCR)* - *Leishmania* spp. total RNA purification was performed from 10^8^ promastigotes using TRIzol (Invitrogen) reagent and treated with DNAse (Invitrogen) for DNA contaminant removal according to the manufacturer’s instructions. The purified RNA was then used in a cDNA synthesis reaction with 500 ng oligo d(T)_12- 18_, using the Superscript III first-strand synthesis system for RT-PCR (Invitrogen). The subsequent *EcmutT* fragment specific amplification was performed using the following primers 5´-GAATTCCCGGACAGGCATATAA-3´ (forward) and 5´-CATTAAGACCGACTAGCGACATC-3´ (reverse). The control of RT-PCR was processed in the same conditions as the samples but without reverse transcriptase enzyme. The *DNA polymerase I alpha catalytic subunit* constitutive gene from *L. braziliensis* (*LbrM.16.1600*) was used to normalise the amount of sample analysed. A fragment of 69 bp of the *DNA polymerase I* gene was amplified according described by Moreira et al.^18^ Both pair of primers *EcmutT* and *DNA polymerase I* amplified fragments of 115 and 69 bp respectively, in all *Leishmania* samples analysed. PCR was carried out in a ﬁnal volume of 20 µL of reaction mixture containing 10 pmol of forward and reverse primers, 1x Supermix High Fidelity reaction mix (Invitrogen) and 10 ng of template cDNA. The PCR conditions were as follows: an initial denaturation step at 95ºC for 5 min followed by 30 cycles of denaturation at 95ºC for 30 s, annealing at 60ºC for 30 s and extension at 72ºC for 30 s. Following amplification, 6 µL of each PCR product was electrophoresed in a 6% polyacrylamide gel and silver stained.

*Susceptibility assays of L. braziliensis and L. infantum clonal lines to Sb* ^*III*^ *and H*_*2*_ *O*_*2*_ *-* Promastigotes of wild-type *L. braziliensis* and *L. infantum* clonal lines non-transfected or transfected with the constructs pIR1BSD (empty vector) or pIR1BSD-*EcmutT* were submitted to Sb^III^ (Sigma-Aldrich, St. Louis, MO, USA) susceptibility tests. Parasites were incubated in M199 medium at 2 x 10^6^ cells/mL into 24-well plates in the absence or presence of several concentrations of Sb^III^ (0.3 to 149.9μM) for 48 h that correspond to log phase of growth. The effective concentration required to decrease growth by 50% (EC_50_) was determined using a model Z1 Coulter Counter (Beckman Coulter, Fullerton, CA, USA). EC_50_ values were determined from at least three independent measurements performed in triplicate, using the linear interpolation method.^19^

To test the survival to H_2_O_2_ (Sigma-Aldrich), parasites were incubated in PBS at 2 x 10^6^ cells/mL into 15 mL tubes in the absence or presence of different concentrations of H_2_O_2_ (100 to 700 μM) for 20 min at 26^o^C. Subsequently the parasites were washed in PBS and incubated in M199 medium. The survival rate of the cultures was determined by counting the number of live parasites during 4, 24, 48, 72, 96, 120, 144 and 168 h of growth after exposure to H_2_O_2_. The parasite number was determined in a cytometry chamber using the erythrosine vital stain to differentiate living and dead cells. In addition, the parasite number was also determined using a model Z1 Coulter Counter (Beckman Coulter) and at least three independent measurements were performed in triplicate.

*Cell cycle analysis* - In order to investigate the effect of H_2_O_2_ treatment on the cell cycle of nontransfected and transfected parasites with the *EcmutT* gene, these parasites incubated in PBS containing 350 μM H_2_O_2_ for 20 min at 26ºC, washed in PBS and incubated in M199 medium during 24 and 72 h. It is important to remove the H_2_O_2_ from medium, since that it may to generate others components which may oxide in the medium. Subsequently, the parasites were washed once with PBS, fixed overnight in 70% ethanol (v/v) and incubated at 37ºC for 30 min in a PBS solution containing 10 μg/mL of propidium iodide (PI) and RNAse A (Invitrogen). The DNA content of PI-incorporated cells was analysed in a FACScan flow cytometer (Becton Dickinson Biosystems) using FloJo-V10^TM^ software. Each experiment was performed at least three times in triplicate.

*Activation percentage of gammaH2A by Western blotting* - For Western blotting assays, exponentially grown *L. braziliensis* and *L. infantum* promastigotes were incubated in PBS containing 350 μM H_2_O_2_ for 20 min, washed in PBS and incubated in M199 medium during 30 min and 4 h and then submitted to protein extraction. Cells were washed and resuspended in sodium dodecyl sulphate (SDS) gel-loading buffer [100 mM Tris-HCl (pH 6.8), 200 mM dithiothreitol, 4% SDS, 0.2% bromophenol blue, 20% glycerol], and boiled for 10 min, generating total extract. Proteins were separated on a 15% SDS polyacrylamide gel, blotted (1 h, 300 mA) onto nitrocellulose membranes, and incubated with rabbit polyclonal anti-γH2A (1:3,000) (kindly provided by Dr Richard McCulloch’s lab) or mouse monoclonal anti-alpha-tubulin (1:5,000) (Abcam, Cambridge, UK) antibodies, during 12 h at 4ºC in the blocking solution. The blots were washed twice and incubated with horseradish peroxidase-conjugated anti-rabbit or anti-mouse IgG antibodies (1:5,000) (GE Healthcare) for 1 h at room temperature. After incubation, the membranes were washed, incubated with ECL Plus chemiluminescent substrate (GE Healthcare) and revealed by ImageQuant LAS 4000 (GE Healthcare). The intensity of the bands was analysed using the software GelAnalyzer 2010 (gelanalyzer.com).

*Analysis of replication stress through the BrdU native detection assay* - For detection of long fragments of single strand DNA (ssDNA), characteristics of replication stress, exponentially growing promastigote parasites were incubated in PBS containing 350 μM H_2_O_2_ and 100 µM of 5-bromo-2’-deoxyuridine (BrdU) for 1 h to allow its incorporation into DNA. Thus, all the samples were harvested by centrifugation (3000 *g*, 5 min), washed using PBS, and fixed using 4% paraformaldehyde diluted in PBS for 10 min at room temperature. Parasite cells were then deposited and spread out onto slides pre-treated with 0.1% poly-L-lysine. Next, parasites cells were washed with PBS, and permeabilised with 0.2% Triton X-100 for 10 min at room temperature. To ensure all cells incorporated BrdU, aliquots of each sample analysed were subjected to DNA denaturation using 2.5 M HCl for 20 min. Then, all samples were washed and BrdU was detected (when accessible) using α-BrdU-rat antibody (Abcam) diluted 1:250 in blocking solution [3% bovine serum albumin (BSA) (w/v) in PBS] for 3 h at room temperature, followed by incubation for more 1 h with Alexa Fluor 555-conjugated anti-rat antibody (Thermo Scientific) diluted 1:1,000 in blocking solution. After that, the slides-containing-cells were washed repeatedly (three times) with PBS. ProLong^®^ Diamond Antifade Mountant with DAPI (Life Technologies) was used to be anti-fade mounting solution and to stain organelles containing DNA (*i.e*., nucleus and kinetoplast). For these experiments, images were captured using Axio Imager fluorescence microscope coupled with a digital camera (Axiocam 503 mono), and were analysed using Zen software 2.6 (blue edition). The measurement of the parasites showing presence of ssDNA foci was made using ImageJ software (NIH, version 1.47t).

*Statistical analysis* - The statistical analysis were performed using the GraphPad Prism 5.0 program (GraphPad Software Inc., CA, USA). All experiments were performed at least three times and data have been represented as mean ± standard deviation (SD). Results were analysed for significant differences using analysis of variance (ANOVA) or Student’s *t* test. A p value of less than 0.05 was considered statistically significant.

## RESULTS

**Fig. 1:**
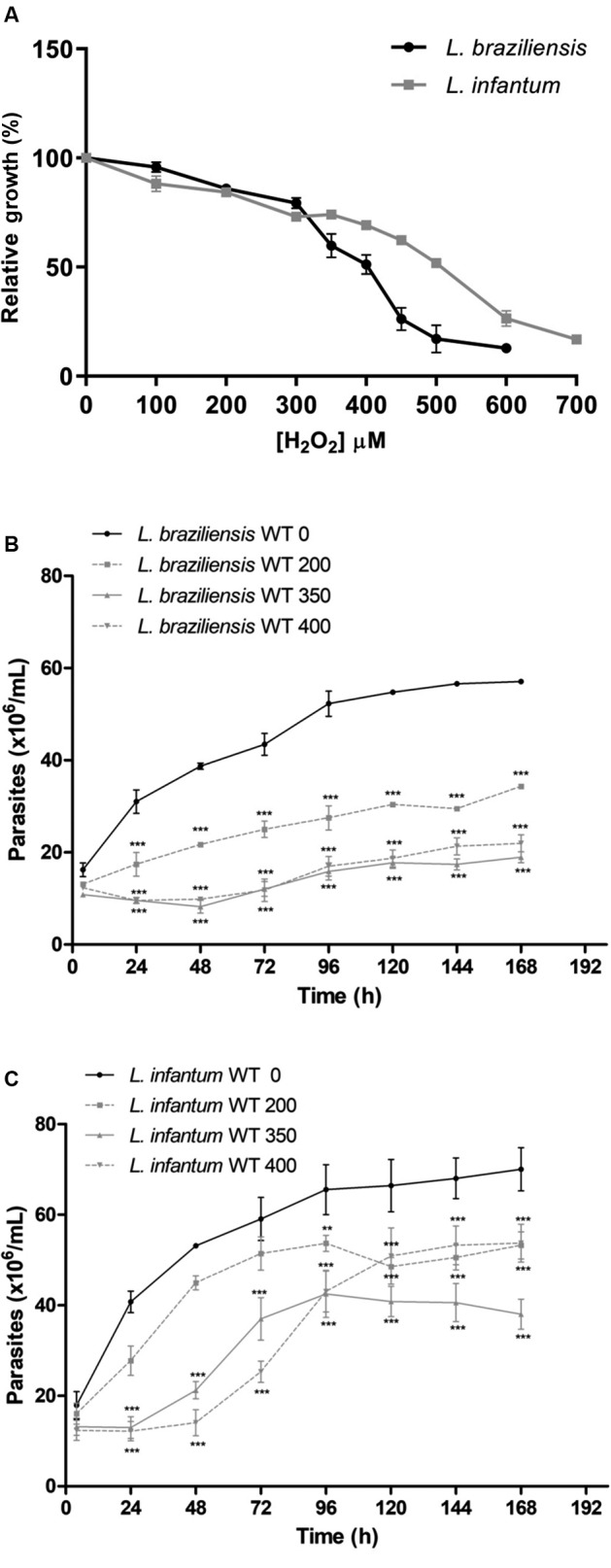
*Leishmania infantum* are more tolerant to oxidative stress generated by H_2_O_2_ than *L. braziliensis.* (A) *L. braziliensis* and *L. infantum* parasites were incubated in the absence or presence of H_2_O_2_ (0 to 700 µM) in phosphate-buffered saline (PBS) for 20 min, washed in PBS and incubated in M199 medium. The parasite number was determined using a model Z1 Coulter Counter after 48 h exposure to H_2_O_2_. (B) *L. braziliensis* and (C) *L. infantum* parasites were incubated in the absence or presence of H_2_O_2_ (0, 200, 350 and 400 µM) for 20 min, washed in PBS and incubated in M199 medium. The parasite number was determined during 4, 24, 48, 72, 96, 120, 144 and 168 h after exposure to H_2_O_2_. Mean values ± standard deviations of three independent experiments in triplicate are indicated. The asterisk symbol (*) indicates the significant difference between non-treated parasites and parasites treated with different H_2_O_2_ concentrations and in each time point. Statistical analysis was performed based on analysis of variance (ANOVA) followed by Bonferroni test (*** p < 0.001, ** p < 0.01, * p < 0.05).

*Comparative analysis of L. braziliensis and L. infantum H*_*2*_ *O*_*2*_ *tolerance* - Comparative analysis of tolerance to oxidative stress generated by increased concentration of hydrogen peroxide was evaluated between *L. braziliensis* and *L. infantum* wild-type species (Fig. 1). Firstly, we evaluated the susceptibility these parasites after 48 h exposure to different concentrations of H_2_O_2_ (100 to 700 µM) (Fig. 1A). Interestingly, *L. infantum* (EC_50_: 505 +/-12.0 μM) is more tolerant to exogenous H_2_O_2_ than *L. braziliensis* (EC_50_: 395 +/- 11.5 μM). In order to better compare the action of H_2_O_2_ in the parasite growth, we treated both *Leishmania* species with the same concentrations of H_2_O_2_ (200, 350 and 400 μM) and verified the parasite growth during 4 to 168 h (Fig. 1B-C). Higher concentrations of H_2_O_2_ (350 µM and 400 µM) are very toxic to *L. braziliensis* and significantly interfere in the parasite growth (Fig. 1B). In contrast, *L. infantum* survive/proliferate to different H_2_O_2_ concentrations and parasites treated with 200, 350 and 400 µM H_2_O_2_ return the growth after 48, 72 and 96 h, respectively after treatment (Fig. 1C).

The parasites growth curves determined during 4 to 168 h after exposure to H_2_O_2_ (Fig. 1B-C), show that at 350 μM H_2_O_2_, *L. infantum* is able to grow again after 48 h exposure to H_2_O_2_ and *L. braziliensis* remains stopped after 48 h, revealing differences between the two species of *Leishmania*. Then the concentration of 350 μM H_2_O_2_ was chosen to analyse cell cycle progression, DNA damage and replication stress generation.

**Fig. 2:**
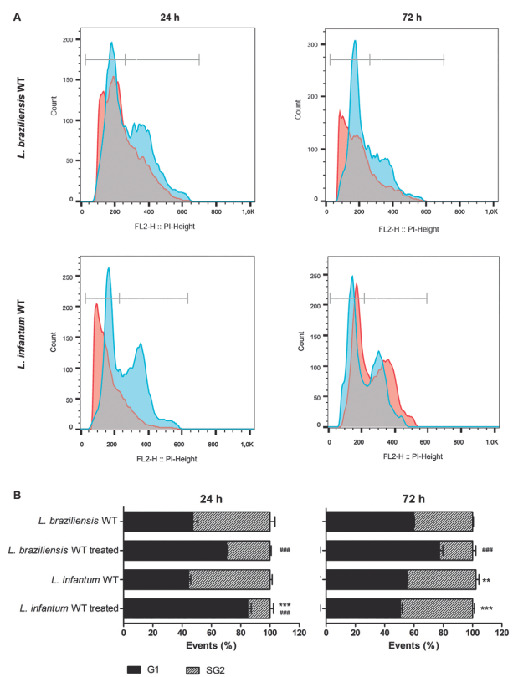
cell cycle comparative analysis by flow cytometry of the *Leishmania braziliensis* and *L. infantum* after exposure to H_2_O_2_. The parasites were treated with 350 μM H_2_O_2_ in phosphate-buffered saline (PBS) for 20 min, washed in PBS and incubated in M199 medium by 24 and 72 h. The data represent at least three independent experiments performed in triplicate. (A) Representative histograms showing the DNA content of *L. braziliensis* and *L. infantum*. Peaks in light blue represent non-treated parasites and peaks in red treated with H_2_O_2_. (B) Quantitative analysis of parasites in each phase after 24 h or 72 h of incubation with or without H_2_O_2_. The asterisk symbol (*) indicates the significant difference between *L. braziliensis* and *L. infantum*, treated with H_2_O_2_ or not. The hash symbol (^#^) indicates the significant difference between treated and non-treated parasites of the same sample. Statistical analysis was performed based on analysis of variance (ANOVA) followed by Bonferroni test (*** p < 0.001, ** p < 0.01, * p < 0.05; or ^###^ p < 0.001).

*Comparative cell cycle analysis of L. braziliensis and L. infantum* - We investigated cell cycle progression of wild-type *L. braziliensis* and *L. infantum* species parasites after 20 min treatment with 350 µM H_2_O_2_ and incubation in M199 medium during 24 and 72 h. After this period, DNA content of propidium iodide-stained cells was analysed in a FACScan flow cytometer. Comparative analysis between *L. braziliensis* and *L. infantum* species (Fig. 2), showed that in the presence of H_2_O_2_ *L. infantum* presented a cell cycle arrest in G1 phase after 24 h, and, after 72 h they restored the normal growth ratio. At 24 h, only 18% of treated *L. infantum* parasites progress in the cell cycle, passing through S and G2 phases, whereas at 72 h 50% of these parasites are in the S and G2 phases showing similar profile compared to untreated parasites (Fig. 2B). *L. infantum* parasites can survive and restore growth after 72 h (Fig. 2B).

On the other hand, *L. braziliensis* has a different cell cycle profile, with little distinction between S and G2 phases. Treated parasites present less cells at S and G2 phases when compared with the non-treated parasites at 24 h and at 72 h, however, *L. braziliensis* is not arrested in G1 phase as *L. infantum* was at 24 h. On the other hand, 72 h after H_2_O_2_ treatment, only 22% of the *L. braziliensis* parasites continue to progress in the cell cycle, passing through S and G2 phases (Fig. 2B). Those results show that *L. braziliensis* was not able to restore growth 72 h after H_2_O_2_ treatment.

*Leishmania spp. cell line expressing EcmutT gene* - Multiple sequence alignment of the *mutT* amino acid sequences from *E. coli* and from *Trypanosoma cruzi*, *L. braziliensis* and *L. infantum* showed that elements for an enzyme from the nudix superfamily were identified in these *Leishmania* ssp. sequences [Supplementary data (Fig. 1)]. This is a family of enzymes that displays great catalytic versatility,^13^ which could indicate as candidate, but they are not necessarily a MutT pyrophosphohydrolase. Further studies need to be performed to confirm these data. The identity between the MutT amino acid sequences of *L. braziliensis* (LbrM.35.0380) and *L. infantum* (LinJ.36.0320) compared to *T. cruzi* TcMTH (AGM37761.1) was 43 and 46%, respectively (data not shown). It is important to highlight that Aguiar et al.^20^ characterised the MutT gene in *T. cruzi* (TcMTH) and generated *T. cruzi* parasites heterologously expressing *E. coli* MutT or overexpressing the TcMTH enzyme. The authors observed the overexpression of the TcMTH gene in *T. cruzi* caused the same phenotypes observed when they expressed the *E. coli* MutT heterologous gene.

**Fig. 3:**
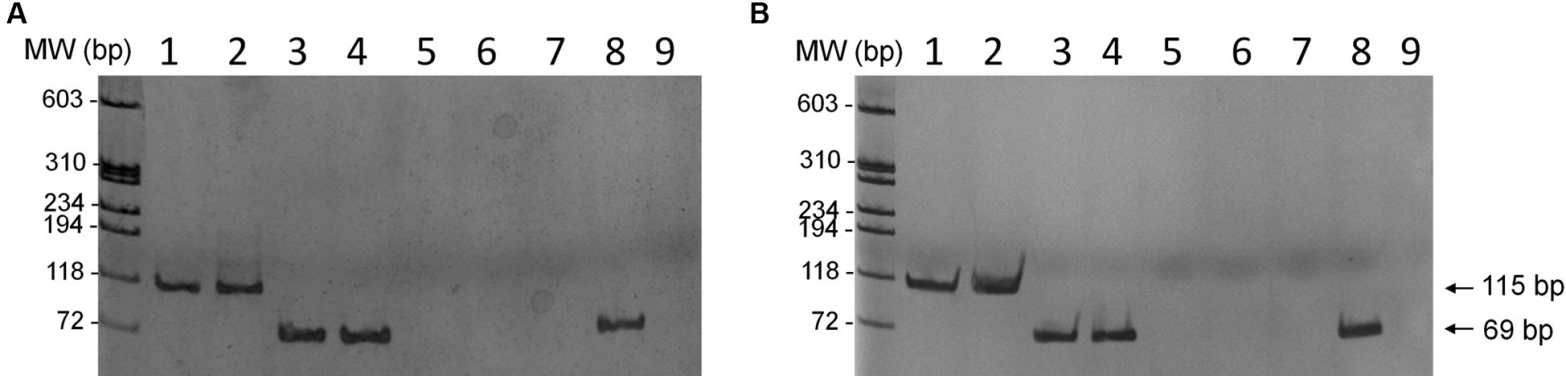
reverse transcription polymerase chain reaction (RT-PCR) of *Leishmania braziliensis* (A) and *L. infantum* (B). RT-PCR was performed to confirm expression of the EcmutT gene in the cDNA of parasites transfected with pIR1BSD-EcmutT construct: clones 16 and 24 from *L. braziliensis* (lanes 1 and 2 - A) and clones 1 and 13 from *L. infantum* (lanes 1 and 2 - B). Electrophoresis was performed on a 6% polyacrylamide gel stained with silver nitrate. The expected 115 bp fragment of EcmutT gene was amplified in both clone lines of *Leishmania* transfected with pIR1BSD-EcMutT (lanes 1 and 2). DNA polymerase was used as quantitative control of RT-PCR reaction, showing fragment of 69 bp (lanes 3 and 4). As control of RT-PCR was used samples of mRNA without the reverse transcriptase enzyme (lanes 5 and 6), showing absence of amplification for both EcMutT and DNA polymerase genes. As control also was used genomic DNA of wild-type non-transfected parasites presenting only amplification of the DNA polymerase gene (lane 8) and not EcmutT gene (lane 7). Negative control of reaction without template (lane 9). The molecular weight markers were derived from Φ 174 DNA digested with *Hae*III (Promega).

Wild-type *L. braziliensis* and *L. infantum* lines were transfected with the constructs containing the *mutT* gene from *E. coli* (pIR1BSD-*EcmutT*) to generate parasites expressing the EcMutT enzyme, and also with an empty vector (pIR1BSD) as a control. Linearisation of this vector allowed integration of the constructs into the ribosomal small subunit locus.^21^ To confirm the transfection, genomic DNA from the transfected clones was subjected to PCR assays using specific primers for the *BSD* gene, which confers resistance to blasticidin. It was observed that all blasticidin-resistant clones (eight clones from each sample) showed a fragment of 399 bp, which corresponds to BSD marker (data not shown). The heterologous expression of *E. coli mutT* gene in the *L. braziliensis* and *L. infantum* cell lines was confirmed through RT-PCR (Fig. 3). The expected 115 bp fragment of the *EcmutT* gene was amplified from the cDNA of parasites transfected with pIR1BSD-EcmutT construct showing the presence of transcript of *EcmutT* gene in the clones of both *Leishmania* species analysed. A fragment of 69 bp of the *DNA polymerase I alpha* constitutive gene, used as control of the assay, was amplified in all samples analysed, except those without reverse transcriptase (RT-PCR negative control).

As the EcMut protein expression was not evaluated, it can not exclude the possibility that the heterologous expression of *E. coli* MutT enzyme could be being differentially expressed in *L. braziliensis* and *L. infantum.*

*The ectopic expression of E. coli MutT does not interfere in parasite growth or in susceptibility to trivalent antimony (Sb* ^*III*^ *)* - To verify whether the expression of *E. coli mutT* gene alter the growth of parasites, promastigotes from *L. braziliensis* and *L. infantum* transfected with the constructs pIR1BSD (empty vector), pIR1BSD-EcMutT and untransfected parasites were incubated in M199 medium during 4 to 168 h [Supplementary data (Fig. 2)]. The growth curves of the *EcmutT*-expressing lines from *L. braziliensis* and *L. infantum* were very similar to their respective wild-type non-transfected parasites. The EcMutT heterologous expression did not alter *L. braziliensis* and *L. infantum* growth.

We also investigated whether the expression of *EcmutT* gene contributes to antimony resistance phenotype in *Leishmania*. For this, *E. coli* MutT-expressing *L. braziliensis* and *L. infantum* clonal lines and non-transfected parasites were incubated with different Sb^III^ concentrations during 48 h [Supplementary data (Fig. 3)]. The EC_50_ was determined by counting the number of parasites that grew in the absence or presence of this drug. EcMutT-expressing *L. braziliensis* and *L. infantum* lines did not show an increase in resistance towards Sb^III^, since that the Sb^III^ EC_50_ of these lines were very similar to respective wild-type non-transfected parasites. The results revealed that the Sb^III^ EC_50_ of wild-type *L. braziliensis* line was 2.66 (± 0.22) μM, and the *EcmutT* expressing clones 16 and 24 were 3.04 (± 0.26) μM and 2.98(± 0.21) μM, respectively [Supplementary data (Fig. 3A)]. Regarding *L. infantum*, we observed that Sb^III^ EC_50_ of wild-type line was 53 (± 0.61) μM and *EcmutT*-expressing clones 1 and 13 were 53 (± 2.34) μM and 55 (± 2.7) μM, respectively [Supplementary data (Fig. 3B)]. It is important to mention that the pIR1-BSD vector did not interfere in the Sb^III^ susceptibility since no difference in Sb^III^ EC_50_ was observed between parasites non-transfected and transfected with empty vector (data not shown).

**Fig. 4:**
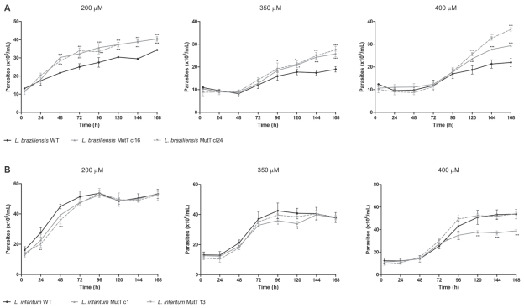
EcMutT heterologous expression increases the resistance to H_2_O_2_ in *Leishmania braziliensis* (A) but not in *L. infantum* (B). Wild-type (WT) and transfected with pIR1-BSD-EcMutT (MutT) *L. braziliensis* (Lb) and *L. infantum* (Li) parasites were incubated with H_2_O_2_ (200, 350 and 400 µM) in phosphate-buffered saline (PBS) for 20 min, washed in PBS and incubated in M199 medium. The parasite number was determined using a model Z1 Coulter Counter during 4, 24, 48, 72, 96, 120, 144 and 168 h after exposure to H_2_O_2_. Mean values ± standard deviations of three independent experiments in triplicate are indicated. The asterisk symbol (*) indicates the significant difference between wild-type parasites and those transfected with EcMutT, treated with each H_2_O_2_ concentration and in each time of growth. Statistical analysis was performed based on analysis of variance (ANOVA) followed by Bonferroni test (*** p < 0.001, ** p < 0.01, * p < 0.05).

*Tolerance of EcMutT expressing Leishmania spp. lines to hydrogen peroxide (H*_*2*_ *O*_*2*_ *)* - We also investigated whether the expression of *EcmutT* gene alter *Leishmania* response to oxidative stress generated by H_2_O_2_. Comparative analysis showed that both EcMutT expressing clones 16 and 24 from *L. braziliensis* were more resistant to oxidative stress generated by H_2_O_2_ compared to untransfected wild-type parasites (Fig. 4A) or parasites transfected with empty vector (data not shown). At 200 µM H_2_O_2_, *L. braziliensis* expressing EcMutT restore their growth after 48 h of incubation. Higher concentrations of H_2_O_2_ (350 µM and 400 µM) are toxic to *L. braziliensis*, but the *EcmutT*-expressing parasites present better growth than wild-type line (Fig. 4A).

We also determined the H_2_O_2_ EC_50_ for different clones incubated in the presence of several concentrations of H_2_O_2_ for 48 h and the results revealed that the H_2_O_2_ EC_50_ of wild-type *L. braziliensis* line was 395 μM, and the *EcMutT* expressing *L. braziliensis* lines clones 16 and 24 were 456 and 443 μM, respectively. These data suggest that MutT is involved in the protection against oxidative stress generated by H_2_O_2_ in *L. braziliensis.*

On the other hand, *EcmutT*-expressing *L. infantum* lines did not alter the H_2_O_2_ susceptibility, since that the parasites present similar growth curve compared to untransfected wild-type parasites (Fig. 4B). However, *L. infantum* clone 1 has its resistance decreased at 400 µM H_2_O_2_ during period of 72 to 168 h after exposure to H_2_O_2_. The same was not observed for *L. infantum* clone 13 that presented similar growth curve compared to wild-type parasites in all H_2_O_2_ concentrations evaluated.

Testing different H_2_O_2_ concentrations for 48 h we observed that the H_2_O_2_ EC_50_ of wild-type *L. infantum* line was 505 μM, and the *EcMutT-*expressing *L. infantum* lines clones 1 and 13 were 499 and 490 μM, respectively. Thus, the results indicated that MutT is not important in the protection against the oxidative stress generated by H_2_O_2_ in *L. infantum*.

**Fig. 5:**
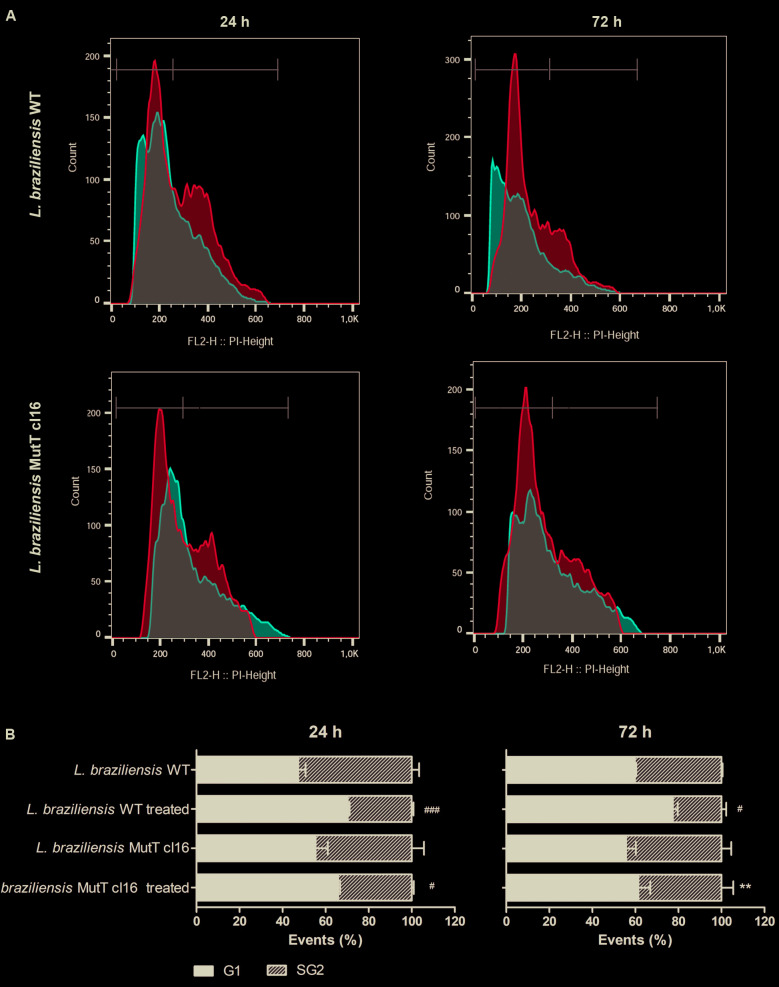
cell cycle comparative analysis by flow cytometry of the wild-type (WT) and clonal lines transfected with pIR1-BSD-EcMutT (EcMutT clone 16) *Leishmania braziliensis* (Lb) parasites after exposure to H_2_O_2_. The parasites were treated with 350 μM H_2_O_2_ in phosphate-buffered saline (PBS) for 20 min, washed in PBS and incubated in M199 medium by 24 and 72 h. The data represent at least three independent experiments performed in triplicate. (A) Representative histograms showing the DNA content of *L. braziliensis* WT and clonal lines. Peaks in light blue represent non-treated parasites and peaks in red treated with H_2_O_2_. (B) Quantitative analysis of parasites in each phase after 24 h or 72 h of incubation with or without H_2_O_2_. The asterisk symbol (*) indicates the significant difference between *L. braziliensis* WT and clonal lines, treated with H_2_O_2_ or not. The hash symbol (^#^) indicates the significant difference between treated and non-treated parasites of the same sample. Statistical analysis was performed based on analysis of variance (ANOVA) followed by Bonferroni test (** p < 0.01; * p < 0.05; ^###^ p < 0.001, ^##^ p < 0.01, ^#^ p < 0.05).

*Cell cycle analysis of EcmutT expressing L. braziliensis* - Comparative analysis of cell cycle between wild-type and *EcmutT*-expressing *L. braziliensis* after H_2_O_2_ treatment showed that at 24 h the cell cycle profile was similar between these parasites (Fig. 5). On the other hand, at 72 h after H_2_O_2_ treatment, while 22.6% of wild-type *L. braziliensis* are in the S and G2 phases, 38.6% of the *EcmutT*-expressor clone 16, continue to progress in the cell cycle, passing through S and G2 phases (Fig. 5B), restoring the normal growth ratio. At 72 h after H_2_O_2_ exposure, different of wild-type *L. braziliensis*, the cell cycle profile of EcMutT-expressor clone 16 is very similar to untreated parasites. These results show that the expression of the *EcmutT* gene protects *L. braziliensis* parasites against H_2_O_2_ effect. No difference in the cell cycle profile was observed between parasites non-transfected and transfected with empty vector (data not shown).

**Fig. 6:**
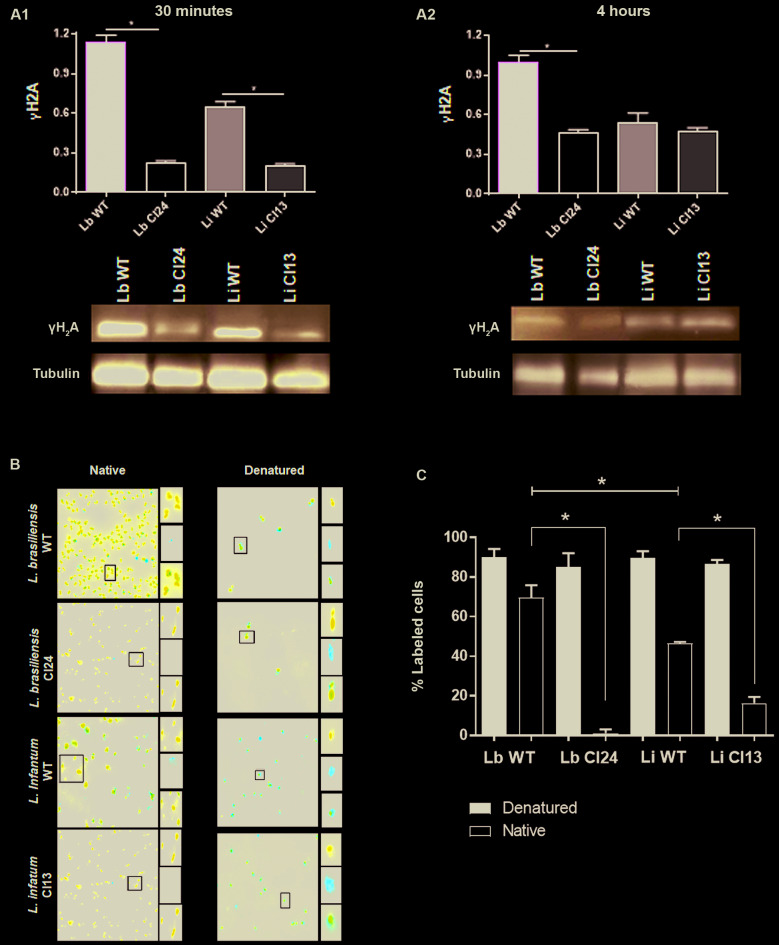
EcMutT expression reduces replication stress in *Leishmania braziliensis* and *L. infantum*. (A) Cells were treated with 350 μM H_2_O_2_ in phosphate-buffered saline (PBS) for 20 min, washed in PBS and incubated with M199 during 30 min and 4 h and then submitted to protein extraction and immunoblotting assays for γH2A. Proteins were separated by electrophoresis on 15% sodium dodecyl sulfate (SDS)-polyacrylamide gel and transferred onto nitrocellulose membranes. The blots were probed with rabbit anti-γH2A (1:3,000) (A1, A2) antibody and developed using ECL Plus kit. The membranes were incubated with the anti-α-tubulin monoclonal (1:5,000) antibody for normalisation of the results. Quantification of the bands was done by densitometric analysis using the software GelAnalyzer 2010 (gelanalyzer.com) and the ratios found for each sample analysed are shown in the Fig. (A1, A2). (B) The cells were treated with 350 μM H_2_O_2_ and 100 µM of 5-bromo-2’-deoxyuridine (BrdU) for 1 h to allow its incorporation into DNA. Parasite were washed, fixed and then deposited onto slides pre-treated with 0.1 % poly-L-lysine. To ensure all cells incorporated BrdU, aliquots of each sample analysed were subjected to DNA denaturation using 2.5 M HCl for 20 min. Then, the samples were divided into two groups; native and denatured. BrdU was detected using rat anti- α-BrdU (1:250) antibody followed by incubation with secondary antibody Alexa Fluor 555-conjugated anti-rat (1:1,000). ProLong^®^ Diamond Antifade Mountant with DAPI was used to be anti-fade mounting solution and to stain organelles containing DNA (*i.e*., nucleus and kinetoplast). Images were captured using Axio Imager fluorescence microscope coupled with a digital camera, and analyzed using Zen software (C) Replication stress foci appearance was higher in WT *L. braziliensis* than *L. infantum*, and EcmutT expressing clones of both species decreased significantly the replication stress. The measurement of the parasites showing presence of ssDNA foci (% labeled cells) was made using ImageJ software. Statistical analysis was performed using Student’s *t* test (* p < 0.001).

*Leishmania spp. expressing EcmutT decreased replication stress caused by H*_*2*_ *O*_*2*_ - *Leishmania* spp. has shown sensibility to H_2_O_2_ treatment which results in cell cycle arrest. To investigate this phenotype, we tested the capacity of wild-type (WT) and EcmutT-expressing cell lines to generate the replication stress after H_2_O_2_ treatment. The heterochromatin modification of histone H2A to γH2A is a biomarker of genotoxic stress.^22^ After 30 min of exposition, no difference of γH2A activation was observed between WT and EcMutT parasites of both *Leishmania* species analysed (Lb) and (Li) (Fig. 6A1). Interestingly, 4 h after H_2_O_2_ treatment, *L. braziliensis* EcMutT-expressing clone 24 demonstrated lower activation of γH2A than its parental non-transfected parasites (Fig. 6A2). Analysis of replication stress was performed using the BrdU native detection assay. The presence of BrdU in single strand DNA strongly suggests replication stress.^23^ After 1 h H_2_O_2_ treatment *L. infantum* parasites presented lower replication stress than *L. braziliensis* (Fig. 6B-C). These results are in accordance with the fact that *L. infantum* is more arrested in G1 phase after H_2_O_2_ treatment. The importance of incorporation of 8-oxoG to generate replication stress is evidenced by the fact that EcMutT-expressing clones decreased 68.35% (± 0.5) of replication stress foci in *L. braziliensis* and 30.25% (± 0.15) in *L. infantum* (Fig. 6B-C).

## DISCUSSION

*Leishmania* needs an effective antioxidant defense system since that the parasite is constantly exposed to reactive oxygen species, produced by anti-leishmania drugs or intrinsic host processes. After infection into vertebrate host, *Leishmania* spp. are phagocytised by macrophages, triggering various reactions of the host defense system. A series of factors act during the activating oxidative process, the main one being the lesions in the DNA, which can be caused, most of the times, by the incorporation of 8-oxoG since it is one of the most abundant oxidative lesions.^10^ Therefore, the presence of an enzyme responsible for repairing or even preventing such lesions is essential, as is the case of the MutT enzyme, which can hydrolyze 8-oxo-dGTP to 8-oxo-dGMP present in the pool of nucleotides, preventing the incorporation of oxidised guanine during the DNA replication.^24^ Foti et al.^25^ demonstrated that the oxidation of guanine to 8-oxoG in the nucleotide pool is one of the forms that antibiotics use to generate cell death, so it is very important to have an enzyme sanitising the cell environment.

In this work we compared the tolerance to oxidative stress and the cell cycle of two different *Leishmania* species, *L. infantum* and *L. braziliensis*, that causes different clinical forms of disease: visceral leishmaniasis or cutaneous leishmaniasis, respectively. We observed that *L. braziliensis* species is more susceptible to hydrogen peroxide when compared to *L. infantum*. Interestingly, *L. braziliensis* also are more susceptible to Sb^III^ than *L. infantum*.^16^ Surface glycoconjugate polymorphisms between *L. infantum* and *L. braziliensis* may possibly cause differences in susceptibility to oxidative stress generated by H_2_O_2_. Lipophosphoglycan (LPG) from the procyclic promastigote form of *L. braziliensis* (M2903) is devoid of side chains, whereas *L. infantum* (PP75) possesses β-glucose residues in approximately one-third of the repeat unit.^26^ This interspecies LPG variation is able to differentially modulate the host cell response.^27^

In order to better understand this difference in oxidative stress susceptibility, we evaluated the cell cycle of these parasites. Cell cycle analysis showed that *L. infantum,* after treatment with H_2_O_2_, remains for a longer time in the G1 phase. By not allowing to progress past the G1 checkpoint, *L. infantum* avoid lesions in DNA caused by 8-oxoG incorporation and generate less replication stress. Thus, the *L. infantum* parasites can survive and restore growth after 72 h. In the other hand, *L. braziliensis* continue to progress in the cell cycle even in the presence of oxidative stress. Despite of this progression through cell cycle, we observe that, *L. braziliensis* is more sensitive to oxidative stress generated by H_2_O_2_. We believe that this occurs because, by allowing progression of the cell cycle, even under unfavorable conditions, the incorporation of 8-oxoG generate replication stress that could generate DNA double-strand break.

To evaluate if EcMutT enzyme is involved in response to oxidative stress, we generated *L. braziliensis* and *L. infantum* parasites heterologously expressing *E. coli* MutT. Interestingly, *L. braziliensis* expressing EcMutT exhibited higher resistance to H_2_O_2_ and presented lower activation of γH2A, a biomarker of genotoxic stress, than non-transfected wild-type parasites. These results show that EcMutT in *L. braziliensis* is involved in the protection against the oxidative stress generated by H_2_O_2_. Analysis of replication stress using the BrdU native detection assay revealed that *L. braziliensis* EcMutT-expressing clone decreased 68.35% of replication stress. As this cell continue to progress to S phase even in the presence of H_2_O_2_, we showed that an excess of MutT enzyme could reduce the quantity of 8-oxoG and, as consequence, reduce the level of replication stress in S phase. These data corroborate with previous results showing that *T. cruzi* expressing *E. coli MutT* gene are more resistant to H_2_O_2_ treatment than wild-type parasites.^20^

In contrast to *L. braziliensis*, expression of *E. coli mutT* gene in *L. infantum* did not alter the susceptibility of parasites to H_2_O_2_. Probably it is due to the fact that *L. infantum* are already more resistant to H_2_O_2_, and, in this way, the excess of MutT is not directly involved in the protection against the oxidative stress generated by H_2_O_2_ in this species. The cell cycle profile of this species confirms these data, since after exposure to H_2_O_2_ this species arrests the growth in G1 phase and the 8oxoG generated could be degraded in this phase before the S phase, so the normal quantity of MutT enzyme in *L. infantum* is sufficient. As the 8-oxoG is less incorporated, *L. infantum* recover the growth faster than *L. braziliensis*, being more resistant to the action of oxidants and can escape faster from the effects of these agents.

In relation to Sb^III^ susceptibility test, the results demonstrated that the expression of EcMutT did not affect the antimony resistance phenotype in both *L. braziliensis* and *L. infantum* species. These data suggest that excess of MutT in *Leishmania* is not directly involved in the protection of parasites from the effect of Sb^III^. In *T. cruzi*, MutT exerts an important defense function against the effects caused by benznidazole,^28^ what was demonstrated when *T. cruzi* overexpressing the *E. coli mutT* gene had a benznidazole resistance index 3-fold higher than the parental parasites.

In other studies, carried out by our group, it was demonstrated that the overexpression of enzymes from antioxidant defense pathway, triparedoxin peroxidase^29^ and iron superoxide dismutase,^30^ increased the resistance of *Leishmania* spp. to trivalent antimony. These data demonstrate the involvement of these antioxidant defense enzymes in the resistance phenotype of *Leishmania* spp. to antimony. Probably, the *E. coli* MutT 8-oxo-dGTPase activity is not directly involved in the Sb^III^ resistance phenotype.

Comparative analysis of cell cycle showed that at 72 h after H_2_O_2_ exposure, the wild-type *L. braziliensis* parasites presented an increase in G1 phase and retention in the S/G2 phases. In contrast, EcMutT-expressor *L. braziliensis* clone 16 recovered their proliferation capacity, restoring the normal growth ratio. These results suggest that the expression of exogenous MutT allows an improved control of oxidised nucleotide incorporation to DNA, preventing lesions that can arise from it, emphasising the importance of 8-oxo-dGTPase hydrolysis. These results agree with previous data showing the importance of oxidised nucleotide clearance in bacteria^25^ and *T. cruzi*.^20^

The results obtained in this work reveal that *L. infantum* species is more resistant to stress generated by H_2_O_2_ than *L. braziliensis* and that *L. braziliensis* and *L. infantum* present important differences in the cell cycle. We hypothesised that there is probably a lower incorporation of 8-oxoG by *L. infantum*, which results in lower number of double-strand breaks in DNA compared to *L. braziliensis*. The excess of MutT enzyme is involved in the protection against oxidative stress in *L. braziliensis*, but not in *L. infantum*.

## References

[1] World Health Organization Leishmaniasis. http://www.who.int/leishmaniasis/disease/en/.Accessed14December2019.

[2] Alvar J, Velez ID, Bern C, Herrero M, Desjeux P, Cano J (2012). Leishmaniasis worldwide and global estimates of its incidence. PLoS One.

[3] Burza S, Croft SL, Boelaert M (2018). Leishmaniasis. Lancet.

[4] Frézard F, Demicheli C, Ferreira CS, Costa MA (2001). Glutathione-induced conversion of pentavalent antimony to trivalent antimony in meglumine antimoniate.. Antimicrob Agents Chemother.

[5] Berman JD, Gallalee JV, Best JM. (1987). Sodium stibogluconate (Pentostam) inhibition of glucose catabolism via the glycolytic pathway, and fatty acid β-oxidation in Leishmania mexicana amastigotes. Biochem Pharmacol..

[6] Wyllie S, Cunningham ML, Fairlamb AH (2004). Dual action of antimonial drugs on thiol redox metabolism in the human pathogen Leishmania donovani.. J Biol Chem.

[7] David SS, O’shea VL, Kundu S (2007). Base-excision repair of oxidative DNA damage. Nature.

[8] Van Loon B, Markkanen E, Hübscher U (2010). Oxygen as a friend and enemy: how to combat the mutational potential of 8-oxo-guanine.. DNA Repair.

[9] Cheng KC, Cahill DS, Kasai H, Nishimura S, Loeb LA (1992). 8-Hydroxyguanine, an abundant form of oxidative DNA damage, causes G----T and A----C substitutions.. J Biol Chem.

[10] Neeley WL, Essigmann JM (2006). Mechanisms of formation, genotoxicity, and mutation of guanine oxidation products. Chem Res Toxicol.

[11] Barnes DE, Lindahl T (2004). Repair and genetic consequences of endogenous DNA base damage in mammalian cells. Annu Rev Genet.

[12] Setoyama D, Ito R, Takagi Y, Sekiguchi M (2011). Molecular actions of Escherichia coli MutT for control of spontaneous mutagenesis. Mutat Res.

[13] Mildvan AS, Xia Z, Azurmendi HF, Saraswat V, Legler PM, Massiah MA (2005). Structures and mechanisms of Nudix hydrolases. Arch Biochem Biophys.

[14] El-Sayed NM, Myler PJ, Bartholomeu DC, Nilsson D, Aggarwal G, Tran AN (2005). The genome sequence of Trypanosoma cruzi, etiologic agent of Chagas disease. Science.

[15] Passos-Silva DG, Rajão MA, Nascimento de Aguiar PH, Vieira-da-Rocha JP, Machado CR, Furtado C (2010). Overview of DNA Repair in Trypanosoma cruzi, Trypanosoma brucei, and Leishmania major. J Nucleic Acids.

[16] Liarte DB, Murta SMF (2010). Selection and phenotype characterization of potassium antimony tartrate-resistant populations of four New World Leishmania species.. Parasitol Res.

[17] Robinson KA, Beverley SM (2003). Improvements in transfection efficiency and tests of RNA interference (RNAi) approaches in the protozoan parasite Leishmania.. Mol Biochem Parasitol.

[18] Moreira DS, Neto RLM, Andrade JM, Santi AMM, Reis PG, Frézard F (2013). Molecular characterization of the MRPA transporter and antimony uptake in four New World Leishmania spp. susceptible and resistant to antimony.. Int J Parasitol.

[19] Huber W, Koella JC (1993). A comparison of three methods of estimating EC50 in studies of drug resistance of malaria parasites. Acta Trop.

[20] Aguiar PHN, Furtado C, Repolês BM, Ribeiro GA, Mendes IC, Peloso EF (2013). Oxidative stress and DNA lesions: the role of 8-oxoguanine lesions in Trypanosoma cruzi cell viability. PLoS Negl Trop Dis.

[21] Goyard S, Beverley SM (2000). Blasticidin resistance: a new independent marker for stable transfection of Leishmania.. Mol Biochem Parasitol.

[22] Damasceno JD, Obonaga R, Silva GLA, Reis-Cunha JL, Duncan SM, Bartholomeu DC (2018). Conditional genome engineering reveals canonical and divergent roles for the Hus1 component of the 9-1-1 complex in the maintenance of the plastic genome of Leishmania.. Nucleic Acids Res.

[23] Silva MS, Muñoz PAM, Armelin HA, Elias MC (2017). Differences in the detection of BrdU/EdU incorporation assays alter the calculation for G1, S, and G2 phases of the cell cycle in Trypanosomatids.. J Eukaryot Microbiol.

[24] Maki H, Sekiguchi M (1992). MutT protein specifically hydrolyses a potent mutagenic substrate for DNA synthesis. Nature.

[25] Foti JJ, Devadoss B, Winkler JA, Collins JJ, Walker GC (2012). Oxidation of the guanine nucleotide pool underlies cell death by bactericidal antibiotics.. Science.

[26] Assis RR, Ibraim IC, Nogueira PM, Soares RP, Turco SJ. (2012). Glycoconjugates in New World species of Leishmania: polymorphisms in lipophosphoglycan and glycoinositolphospholipids and interaction with hosts. Biochim Biophys Acta.

[27] Ibraim IC, Assis RR, Pessoa NL, Campos MA, Melo MN, Turco SJ (2013). Two biochemically distinct lipophosphoglycans from Leishmania braziliensis and Leishmania infantum trigger different innate immune responses in murine macrophages. Parasit Vectors.

[28] Rajão MA, Furtado C, Alves CL, Passos-Silva DG, Moura MB, Schamber Reis BL (2014). Unveiling benznidazole’s mechanism of action through overexpression of DNA repair proteins in Trypanosoma cruzi. Environ Mol Mutagen.

[29] Andrade JM, Murta SMF (2014). Functional analysis of cytosolic tryparedoxin peroxidase in antimony-resistant and-susceptible Leishmania braziliensis and Leishmania infantum lines. Parasit Vectors.

[30] Tessarollo NG, Andrade JM, Moreira DDS, Murta SMF (2015). Functional analysis of iron superoxide dismutase-A in wild-type and antimony-resistant Leishmania braziliensis and Leishmania infantum lines. Parasitol Int.

