# Supplementary material for: The heterologous expression of Escherichia coli MutT enzyme is involved in the protection against oxidative stress in Leishmania braziliensis
Source: Mem Inst Oswaldo Cruz. 2020 Jul 6;115:e190469. doi: 10.1590/0074-02760190469 (PMC7337112; doi:10.1590/0074-02760190469)
Supplement: Supplementary file 1 [file 1678-8060-mioc-115-e190469-s.pdf]

```
CLUSTAL O(1.2.4) multiple sequence alignment


LbrM.31.3320                ---MSRLLEFTFSGGQLRRLEQERMGPGFQVQRLQPSYKPRVVIVSKENVAYVPRTQRQP 57
LinJ.31.3060                ---MSRPLEFAFSGGHLRRLEQERMVPGFQVQRLQPSHKPRVVIAKKEHAGFVPRAQRQP 57
sp|P08337|MUTT_ECOLI        ------------------------------------------------------------ 0
[Trypanosoma                ------------------------------------------MAAMTATVHLL------- 11
LinJ.36.0320                MCCHTSPLRFSYPHTVLRSNR-----------------RRRHMIRATAAAARL------- 36
LbrM.35.0380                ------------------------------------------MIRTAAAAARL------- 11


LbrM.31.3320                NVGWWHHSLCDGLLTVKDLKELIYVGEDQRTGENIFSSMADNLAGPRLSQLAWAPSKDGC 117
LinJ.31.3060                NVAWWHHSLCDGALIVKDLKELIYVGEDPRTGENIFSSMADNLAGRRLSQLIWAPSKDGH 117
sp|P08337|MUTT_ECOLI        ------------------------------------------------------------ 0
[Trypanosoma                ----------------------------------------------PRTTSEWIALV--- 22
LinJ.36.0320                ----------------------------------------------PQSTQGWVPLM--- 47
LbrM.35.0380                ----------------------------------------------PQSAQGWVPLL--- 22


LbrM.31.3320                VFEMSRSDQAAYGLAISLVRWHASNRFCANCGTKTDTTHDVGFSRLCPECR----QQ--R 171
LinJ.31.3060                VLEMARPEQAAYGLAMSLVRWHASNRFCANCGTATDPTRDVGFSRLCPKCR----KQ--H 171
sp|P08337|MUTT_ECOLI        ------------------------------------------------------------ 0
[Trypanosoma                --------QRALPLRLDEL--RMPNHFHLKDI-HTGRCRSHL----QHSMPPHSKRESAV 67
LinJ.36.0320                --------QRALDFPIESV--TFPSHFYLLTV-NKGTVSTVGFSPQKAPVQLTAPRQCAV 96
LbrM.35.0380                --------QQALSFPIESV--AFPSHFCLHTF-SDGSTTTTGFSPQKTPVKLTTPRQCAV 71


LbrM.31.3320                FPQIMPAVLVAVMDGKGNVIL--SQRR----KKSQLLTLLSGFILHGESAEETVRREVEE 225
LinJ.31.3060                FPQLVPAVLVAVLDGKGNVIL--SQRR----KESKMLTLLSGFVLHGESAEETVRREVEE 225
sp|P08337|MUTT_ECOLI        MKKLQIAV-GIIRNENNEIFI---TRRAADAHMANKLEFPGGKIEMGETPEQAVVRELQE 56
[Trypanosoma                LILLSPPV-GFTGKGFQEMCMTLTKRTAKLRHHRSQMSFPGGKVDHGETIIAAAQRETME 126
LinJ.36.0320                LVLLSPA---ATENGFQDMCITLTKRTATMGSHKSEMSFPGGHVDADETLRNAAQRETLE 153
LbrM.35.0380                LVLLSPT---ATGDGFQDMCITLTKRTETVSSHKSEMSFPGGHAEADETPRNAAQREALE 128
                            :  :         .   :: :    *        . : : .*    .*:   :. **  *

LbrM.31.3320                ETGAKVSEVRYIGSQPWPHPYLIMMCYY---AVADASPTLAV----EVAELKSVSWVSKQ 278
LinJ.31.3060                ESGARVSEVRYIGSQPWPYPYLMMMCYY---AVADASPSLVV----DASELEKVMWVCKQ 278
sp|P08337|MUTT_ECOLI        EVGITPQHFSLFEKLEYEFPDRHITL--WFWLVERW-EGEPWGKEGQPGEWMSLVGLNAD 113
[Trypanosoma                EIGIPASSYFVIGTLHPIYSFDGGSKVFPVVAVAES-AVEPVCK--SPVEVASIHYMHLS 183
LinJ.36.0320                EVGLPPSEYKIIGSLTPITMRALDARVTPFVAVATS-PVQPYRA--SPAEVDSIHYLHMS 210
LbrM.35.0380                EVGLLPSEYEIIGSLTPISTKSQHVCVTPFVAVATK-PVQPHRA--SPEEVDSLHYLHLS 185
                            * *   .    : .                  *             .  *  .:  : .

LbrM.31.3320                DVRCALEGRHSD-IKLHG-----PGTTPYAMLK-------------PWVDGEVDDYGRVI 319
LinJ.31.3060                DVRRALEGQHSD-MELRG-----PGTTPYAMLK-------------PWVDGEVDDRGQVT 319
sp|P08337|MUTT_ECOLI        DFPPANEPVIA---KLKRL----------------------------------------- 129
[Trypanosoma                RLLLESERTHCRLIKRHSLTGGMPSYFPCFFASESQAVVCGDVCPTKNTPSIPEDGGLLP 243
LinJ.36.0320                TLLLRASTGHARVIKYRSFTSDRPCFFPCFFASPAQTVVSSAVCRSPKATGVADDCGFDP 270
LbrM.35.0380                TLLLKASTSHARVIKYHSLTSKRPCFFPCFFASPVQMDVSPAVRRSPKAAVVMDDCGFDP 245
                             .    .   .   : :

LbrM.31.3320                KPQSRL------------------------------------------------------ 325
LinJ.31.3060                KPQSQL------------------------------------------------------ 325
sp|P08337|MUTT_ECOLI        ------------------------------------------------------------ 129
[Trypanosoma                MPRENFPGELVWGITAFITCELLVRLSAVLELSHP--SEGDAMGLLRCSSVVARDPECIY 301
LinJ.36.0320                MLPEDFPGELVWGLTSFVTCELVARVAKAIMDAPEASSAAAVNALLTPSNVVARDPLADS 330
LbrM.35.0380                MLPEDFPGELVWGLTSLMTCELVARVAKVIVDAPEANSAAEVNALLIPSNVVARDPLADS 305


LbrM.31.3320                ----- 325
LinJ.31.3060                ----- 325
sp|P08337|MUTT_ECOLI        ----- 129
[Trypanosoma                KENSS 306
LinJ.36.0320                PQ--- 332
LbrM.35.0380                TR--- 307
```

Fig. 1: multiple sequence alignment of the *mutT* amino acid sequences from *Escherichia coli* (P08337), *Trypanosoma cruzi* (AGM37761.1), *Leishmania infantum* (LinJ.31.3060 and LinJ.36.0320) and *L. braziliensis* (LbrM.31.3320 and LbrM.35.0380). The black box encloses the Nudix motif. The asterisks (*) represents the maximum identity between sequences analised; the two point mark (:) represents change of two amino acids; the end point (.) represents change of amino acid residue.

CC BY

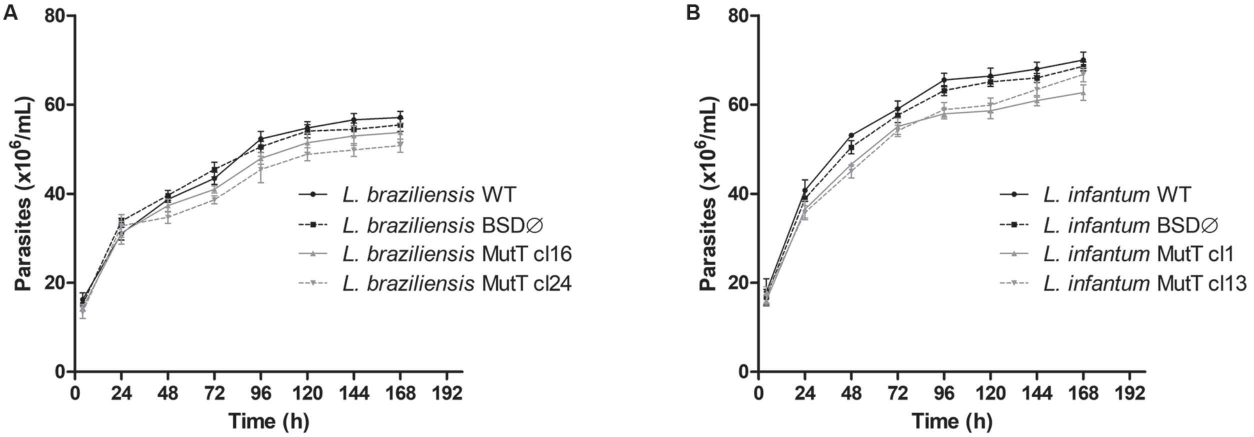

Fig. 2: MutT heterologous expression did not alter *Leishmania braziliensis* (A) and *L. infantum* (B) growth. Wild-type (WT), parasites transfected only with pIR1-BSD plasmid (BSDØ) and clones transfected with pIR1-BSD-MutT were growth in M199 medium and followed for 4 to 168 h until stationary phase. The parasite number was determined using a model Z1 Coulter counter. Experiments were performed three times in triplicate.

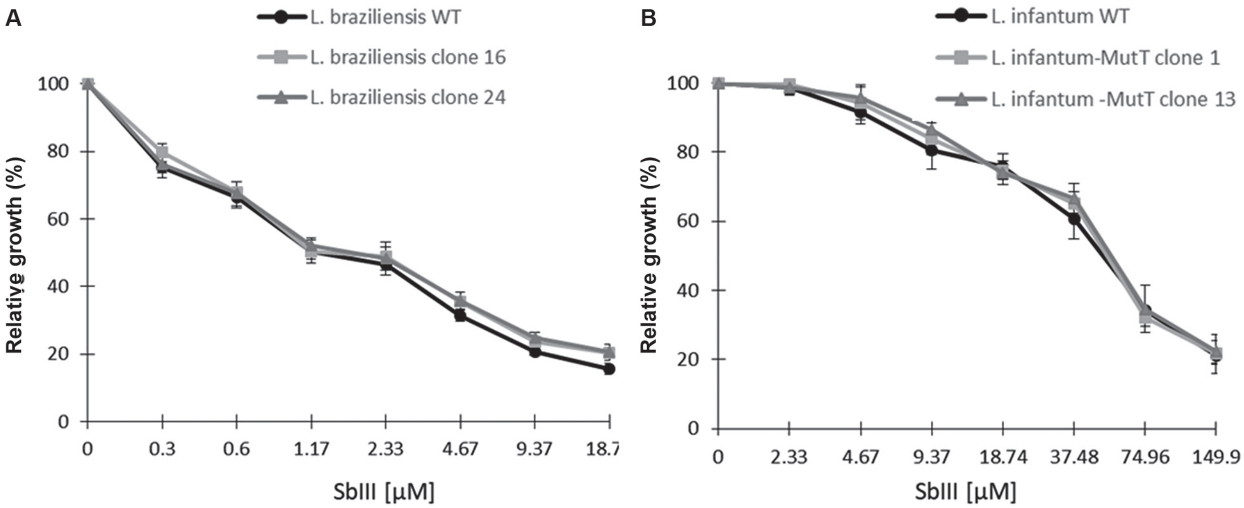

Fig. 3: EcMutT heterologous expression did not affect SbIII susceptibility in *Leishmania braziliensis* (A) and *L. infantum* (B). Susceptibility to SbIII was evaluated in wild-type (WT) and transfected with pIR1-BSD-MutT *L. braziliensis* (A) and *L. infantum* (B) lines. Parasites were incubated in the absence or presence of SbIII (0.3 to 149.9 μM) for 48 h and the percentages of parasites were determined using a model Z1 Coulter Counter. Mean values ± standard deviations of three independent experiments in triplicate are indicated.
